# Gut Microbiota in Human Adults with Type 2 Diabetes Differs from Non-Diabetic Adults

**DOI:** 10.1371/journal.pone.0009085

**Published:** 2010-02-05

**Authors:** Nadja Larsen, Finn K. Vogensen, Frans W. J. van den Berg, Dennis Sandris Nielsen, Anne Sofie Andreasen, Bente K. Pedersen, Waleed Abu Al-Soud, Søren J. Sørensen, Lars H. Hansen, Mogens Jakobsen

**Affiliations:** 1 Department of Food Science, University of Copenhagen, Frederiksberg, Denmark; 2 Department of Infectious Diseases and CMRC, University Hospital Rigshospitalet, Copenhagen, Denmark; 3 Department of Biology, University of Copenhagen, Copenhagen, Denmark; Charité-Universitätsmedizin Berlin, Germany

## Abstract

**Background:**

Recent evidence suggests that there is a link between metabolic diseases and bacterial populations in the gut. The aim of this study was to assess the differences between the composition of the intestinal microbiota in humans with type 2 diabetes and non-diabetic persons as control.

**Methods and Findings:**

The study included 36 male adults with a broad range of age and body-mass indices (BMIs), among which 18 subjects were diagnosed with diabetes type 2. The fecal bacterial composition was investigated by real-time quantitative PCR (qPCR) and in a subgroup of subjects (N = 20) by tag-encoded amplicon pyrosequencing of the V4 region of the 16S rRNA gene. The proportions of phylum *Firmicutes* and class *Clostridia* were significantly reduced in the diabetic group compared to the control group (P = 0.03). Furthermore, the ratios of *Bacteroidetes* to *Firmicutes* as well as the ratios of *Bacteroides*-*Prevotella* group to *C. coccoides*-*E. rectale* group correlated positively and significantly with plasma glucose concentration (P = 0.04) but not with BMIs. Similarly, class *Betaproteobacteria* was highly enriched in diabetic compared to non-diabetic persons (P = 0.02) and positively correlated with plasma glucose (P = 0.04).

**Conclusions:**

The results of this study indicate that type 2 diabetes in humans is associated with compositional changes in intestinal microbiota. The level of glucose tolerance should be considered when linking microbiota with metabolic diseases such as obesity and developing strategies to control metabolic diseases by modifying the gut microbiota.

## Introduction

Type 2 diabetes is a metabolic disease which primary cause is obesity-linked insulin resistance. However, some other factors like mental stress, infection and genetic predisposition might lead to diabetes as well [1]-[4]. Both obesity and diabetes are characterized by a state of chronic low-grade inflammation with abnormal expression and production of multiple inflammatory mediators such as tumor necrosis factor and interleukins [5]. Recent studies based on large-scale 16S rRNA gene sequencing and more limited techniques, based on quantitative real time PCR (qPCR) and fluorescent in situ hybridization (FISH), have shown a relationship between the composition of the intestinal microbiota and metabolic diseases like obesity and diabetes. For example, levels of *Bifidobacterium* significantly and positively correlated with improved glucose-tolerance and low-grade inflammation in prebiotic treated-mice [1], [6]. Furthermore, the development of diabetes type 1 in rats was reported to be associated with higher amounts of *Bacteroides* ssp. [7]. It has been proposed that the gut microbiota directed increased monosaccharide uptake from the gut and instructed the host to increase hepatic production of triglycerides associated with the development of insulin resistance [8].

Several studies on mice models and in humans provided evidence that increase in body weight was associated with a larger proportion of *Firmicutes* and relatively less *Bacteroidetes* [9]-[11]. In accordance with these results, Zhang and coworkers [12] demonstrated that *Firmicutes* were significantly decreased in post-gastric-bypass individuals, and *Prevotellaceae* highly enriched in obese individuals. The differences in microbial composition were explained by an increased capacity of the obesity-associated microbiome to harvest energy from the diet [13]. Controversial data were recently reported by Schwiertz and colleagues [14]. They determined lower ratios of *Firmicutes* to *Bacterodetes* in overweight human adults compared to lean controls. Another study, using weight loss diets, found no proof of the link between the proportion of *Bacteroidetes* and *Firmicutes* and human obesity [15]. Consequently, the composition of obese microbiome is still questionable and more scientific evidence is needed to elucidate the relationship between the gut microbial composition and metabolic diseases.

Most of the published studies describe the differences between gut microbiota in obese compared to lean persons, while type 2 diabetes is generally considered as an attribute to obesity and thus far left behind as the focus of research. The objective of this study was to characterize the composition of fecal microbiota in adults with diabetes type 2 as compared to non-diabetic controls using tag-encoded amplicon pyrosequencing of the V4 region of the 16S rRNA gene and qPCR.

## Results

### Subjects

Subjects with type 2 diabetes (N = 18) and non-diabetic controls (N = 18) were all males at age 31 to 73 years and body mass indices (BMIs) ranging from 23 to 48 (Table 1). The diabetic group had elevated concentration of plasma glucose as determined by a fasting oral glucose tolerance test (OGTT). Subject C17, though having high plasma glucose, was referred to the non-diabetic group based on the measurements of baseline glucose and biochemical analysis of blood samples. The two groups were comparable with regard to their characteristics.

**Table 1:** Characteristics of the diabetic subjects and controls in the study: age, body mass index (BMI) and plasma glucose concentration measured by oral glucose tolerance test (OGTT).

Subjects with diabetes type 2 (N = 18)				Control group (N = 18)			
Subject ID	Age, Years	BMI Kg/m^2^	OGTT glucose mmol/l	Subject ID	Age, Years	BMI Kg/m^2^	OGTT glucose mmol/l
D1	40	24	25.8	C1	34	25	4.8
D2	51	48	27.9	C2	54	27	4.2
D3	69	25	13.3	C3	60	24	3.8
D4	62	27	20.6	C4	68	39	3.5
D5	42	34	20.5	C5	39	31	5.5
D6	72	23	22.1	C6	58	33	4.7
D7	53	23	24.7	C7	55	39	4.6
D8	31	43	20.6	C8	52	24	5.4
D9	54	33	19.0	C9	58	27	5.3
D10	52	25	18.1	C10	72	27	5.1
D11	36	36	8.8	C11	70	29	6.5
D12	64	22	12.1	C12	43	25	3.2
D13	76	27	19.3	C13	59	23	5.2
D14	61	28	15.0	C14	68	28	5.6
D15	60	32	22.0	C15	74	23	4.5
D16	65	27	8.8	C16	65	32	4.2
D17	49	31	17.4	C17	64	24	9.2
D18	73	23	18.7	C18	63	24	4.0
*Mean (s.d.)*	*56 (13)*	*30 (7)*	*18.6 (5.4)*	*Mean (s.d.)*	*59 (11)*	*28 (5)*	*5.0 (1.3)*

### Characterization of the Intestinal Microbiota by Tag-Encoded Pyrosequencing

The total number of reads obtained for 20 subjects by V4 16S rRNA pyrosequencing was 1028955. After applying quality control and trimming we obtained 382229 high quality sequences from diabetic persons and 357782 sequences from healthy controls, accounting for 71.9% of the total reads (Table 2). The number of sequences varied between the subjects from 10521 to 66999 with a mean of 37000 (SD 16062). The average sequence length of trimmed sequences was 234 bp.

**Table 2:** The number of sequences produced and equalized; the number of operational taxonomic units (OTUs) and richness estimates (Chao1) at 3% distance within fecal samples of the diabetic persons (D1-D10) and controls (C1–C10) as determined by pyrosequencing of the V4 region of the 16S rRNA gene.

Sample ID	Sequences produced	Sequences equalized	OTUs	Chao1	Confidence interval (95%)
D1	41607	20000	1416	2487	2286–2736
D2	36644	20000	1617	2868	2650–3133
D3	49213	20000	934	1494	1364–1663
D4	54663	20000	1396	2337	2161–2554
D5	66097	20000	1776	3023	2814–3274
D6	10521	10521	1140	1773	1643–1937
D7	26874	20000	1022	1605	1472–1777
D8	27581	20000	1415	2161	2016–2340
D9	27768	20000	1985	3127	2939–3352
D10	41261	20000	1233	1971	1823–2157
C1	28126	20000	1846	3049	2851–3286
C 2	42207	20000	1516	2452	2279–2665
C3	30701	20000	2473	4269	4010–4571
C4	55678	20000	1411	2122	1981–2298
C5	27204	20000	1079	1689	1555–1861
C6	48719	20000	1595	2527	2360–2730
C7	22794	20000	2254	3769	3535–4047
C8	17080	17080	824	1184	1094–1304
C9	18274	18274	456	655	589–755
C10	66999	20000	1322	1911	1793–2059

**Figure 1:**
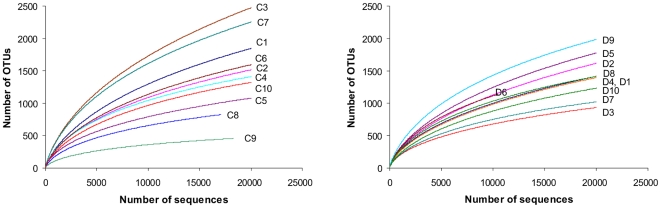
Rarefaction curves. Rarefaction analysis of V4 pyrosequencing tags of the 16S rRNA gene in fecal microbiota from adults with diabetes type 2 (D1–D10) and non-diabetic controls (C1–C10). Sample codes are the same as in Table 1. Rarefaction curves were constructed at 3% distance using RDP release 10 (Pyrosequencing pipelines).

The mean bacterial diversity, as estimated by Chao1 indices from the equalized data sets (Table 2), was not different between the diabetic subjects and the controls, comprising 2287 (SD 587) and 2363 (SD 1113), respectively. However, the variability of Chao1 estimates between the diabetic subjects (Chao1 from 1364 to 2939) was lower compared to the controls (Chao1 from 589 to 4010). As shown by the rarefactions curves, bacterial diversity and richness in diabetic subjects with BMI more than 31 (Figure 1, D2, D5, D8 and D9) was somewhat higher than in lean diabetics, with the means Chao1 of 2785 (SD 436) and 1945 (SD 399), respectively. The same tendency was observed in the control group (Fig. 1, subjects C4, C6, C7 with BMI >31).

**Figure 2:**
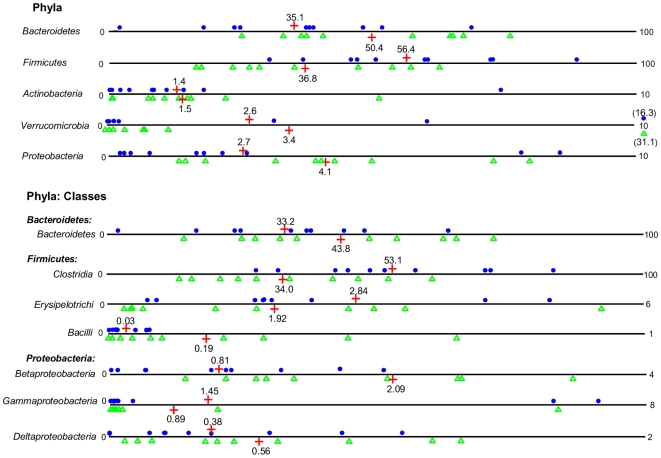
Relative abundances of bacterial phyla and classes. Relative abundances (%) of bacteria were determined in feces from human adults with type 2 diabetes (green triangles, N = 10) and non-diabetic controls (blue dots, N = 10) by pyrosequencing analysis of the V4 region of the 16S rRNA gene. Mean values are denoted by red crosses and numbers. Values out of scale are shown in brackets.

**Figure 3:**
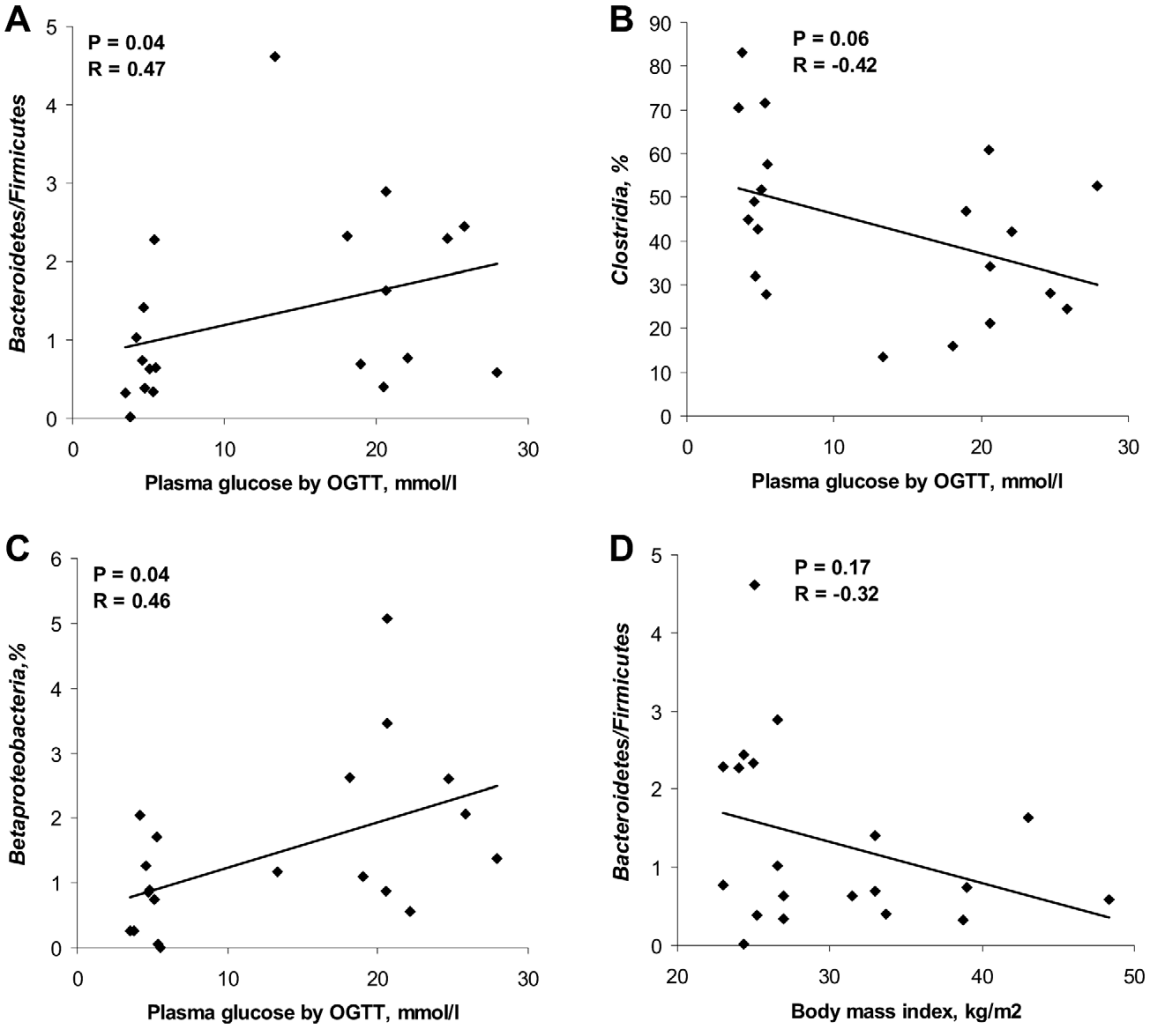
Correlation between OGTT or BMI and bacterial estimates by pyrosequencing. Correlation between OGTT plasma glucose and (A) ratios of *Bacteroidetes* to *Firmicutes*, (B) relative abundance of *Clostridia*, (C) relative abundance of *Betaproteobacteria*. (D) Correlation between body mass indices and ratios of *Bacteroidetes* to *Firmicutes*. The Spearman Rank probability (P) and correlation (R) are shown in the graphs. Bacterial abundances were determined by pyrosequencing of the V4 region of the 16S rRNA gene in fecal bacterial DNA from human adults with type 2 diabetes (N = 10) and non-diabetic controls (N = 10).

Sequences were distributed among 5 bacterial phyla including *Firmicutes* and *Bacteroidetes*, together harboring on average up to 90% of sequences, as well as phyla *Proteobacteria*, *Actinobacteria* and *Verrumicrobia*, each accounting for 1–4% of the sequences (Figure 2). The proportion of *Firmicutes* was significantly higher (P = 0.03) in the controls (mean 56.4%) compared to the diabetic group (mean 36.8%). Accordingly, phylum *Bacteroidetes* and *Proteobacteria* were somewhat but not significantly enriched in the diabetic group. Furthermore, ratios of *Bacteroidetes* to *Firmicutes* correlated positively and significantly with the values of plasma glucose determined by OGTT (R = 0.47, P = 0.04) and negatively, though not significantly, with BMI (R = −0.32, P = 0.17; Figure 3A, D). Relative abundances of *Actinobacteria* and *Verrumicrobia* were not significantly different between the groups (Figure 2).

Phylum *Bacteroidetes* was primarily presented by class *Bacteroidetes* that was on average slightly increased in diabetics (44%) compared to controls (33%; Figure 2). Most of the sequences from *Firmicutes* belonged to the class *Clostridia*, varying from 14 to 72% between the subjects. Other classes included *Erysipelotrichi* and *Bacilli*, accounting for less than 6% and 1% of the sequences, respectively. The proportion of *Clostridia* in diabetics was significantly lower than in controls (P = 0.03; means of 53% versus 34%, respectively) and showed a tendency to decrease with higher levels of plasma glucose (R = −0.42, P = 0.06; Figure 3B). The relative abundance of class *Bacilli* was increased in diabetics at close to significant levels (mean 0.19% versus 0.03% in controls, P = 0.06; Figure 2). Similarly, class *Betaproteobacteria*, belonging to *Proteobacteria*, was highly enriched in subjects with diabetes (mean 2.09% versus 0.81% in controls; P = 0.02) and positively correlated (R = 0.46, P = 0.04) with plasma glucose (Fig. 3C). The relative abundances of bacterial genera (see SI Figure S1) were not significantly different between the groups. However, the proportion of genus *Roseburia* negatively correlated with plasma glucose at levels close to significance (R = −0.52, P = 0.06; data not shown), whereas the opposite trend was observed for the genus *Prevotella* (R = 0.32, P = 0.15; data not shown).

**Figure 4:**
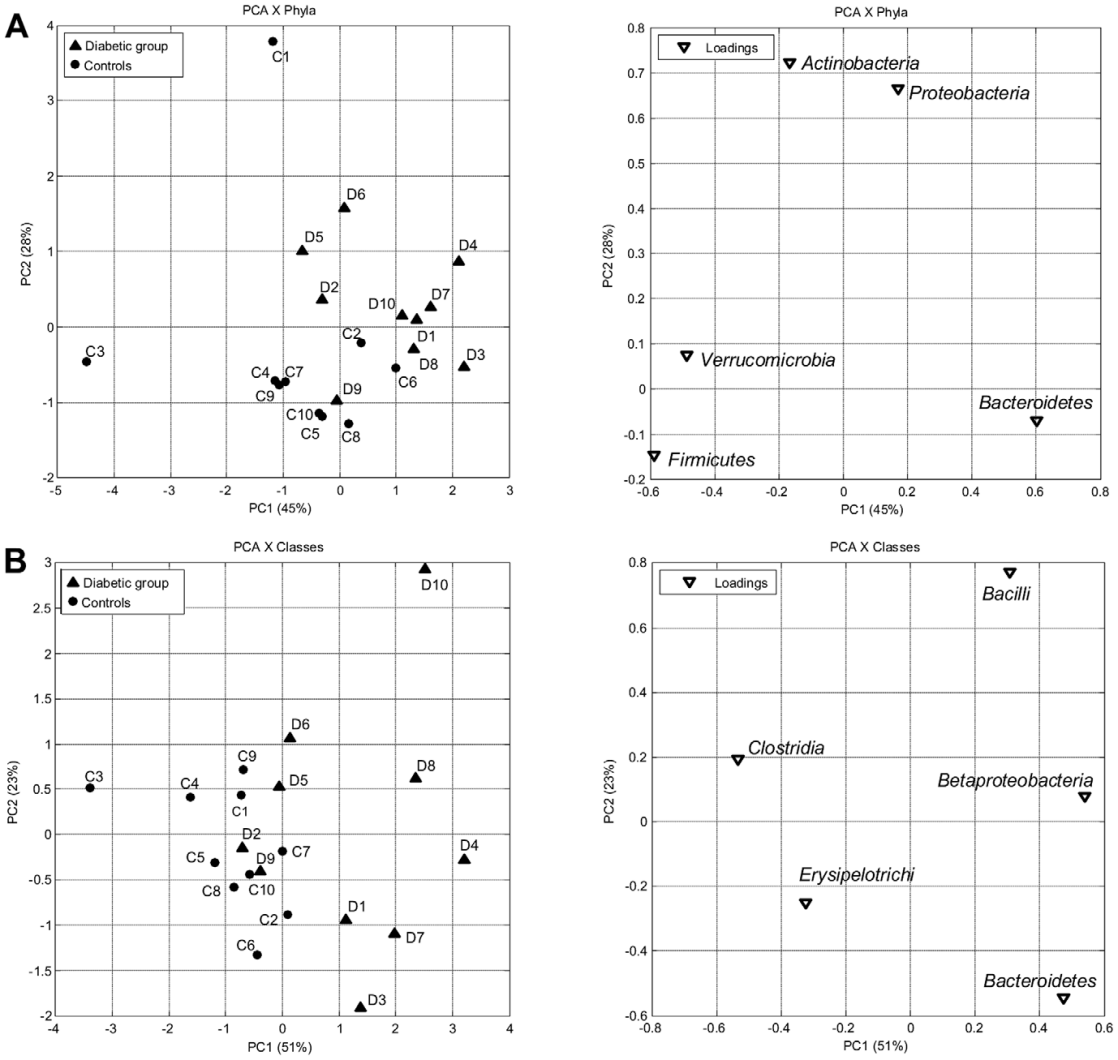
PCA plots of bacterial phyla and classes. PCA plots showing the grouping of human adults with type 2 diabetes (▴, D1–D10) and non-diabetic controls (•, C1–C10) according to the abundances of bacterial phyla (A) and classes (B) in the fecal bacterial DNA as determined by pyrosequencing of the V4 region of the 16S rRNA gene.

As seen from principal component analysis (PCA), a separation between the diabetic group and the control group at the level of bacterial phyla could be best observed from PC1 and PC2 (Figure 4A; 45% and 28% of explained variance, respectively). It was attributed to *Proteobacteri*a and *Actinobacteria* in the second direction (PC2) in combination with *Bacteroides* versus *Firmicutes* and *Verrumicrobia* in the first direction (PC1). At the level of bacterial classes the variation was predominantly linked to a higher positive score for *Betaproteobacteria*, *Bacteroidetes* and *Bacilli* in the diabetic group, versus *Clostridia* and *Erysipelotrichi* in the control group (Figure 4B). The PCA plots of bacterial families generally indicated low levels of systematic variation where only 20–30 percent of the variation was explained by successive PCs, and no distinct clustering was observed (see SI Figure S2A). No association of the diabetic group with particular bacterial genera was apparent. Nevertheless, some of the diabetic persons (see SI Figure S2B, subjects D1, D3, D4, D6, D8, D10) could be discriminated from most of the controls by genus *Prevotella* versus the combination of genera *Lachnospiraceae IS*, *Roseburia* and *Subdoligranulum* (PC1 50%). PCA of multiple genera showed no clear grouping of the subjects with diabetes probably due to the high individual differences masking the systematic variation (data not shown).

### Quantification of the Intestinal Microbiota by qPCR

**Figure 5:**
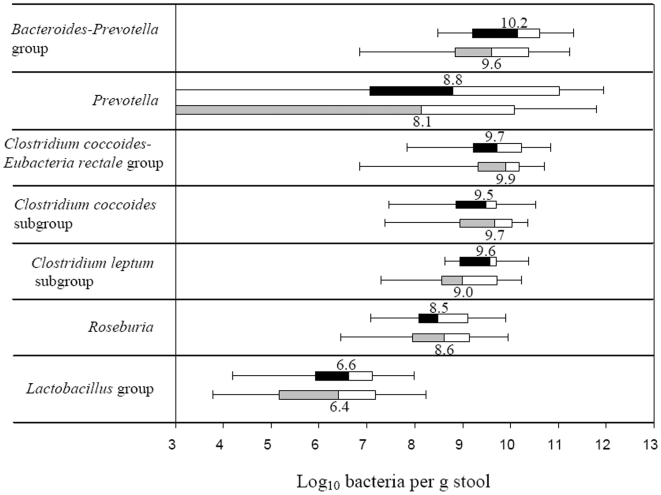
Box-and-whisker plots of bacterial groups quantified by qPCR. Bacterial groups quantified by SYBR Green qPCR and expressed as Log_10_ bacteria per g stool in human adults with type 2 diabetes (black and white boxes; N = 18) and non-diabetic controls (grey and white boxes; N = 18). The median counts are presented by numbers. Boxes show the upper (75%) and the lower (25%) percentiles of the data. Whiskers indicate the highest and the smallest values.

**Figure 6:**
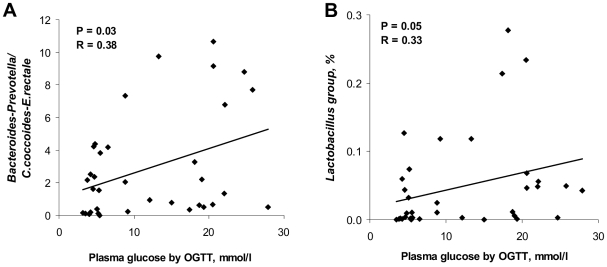
Correlation between OGTT and bacterial estimates by qPCR. Correlation between OGTT plasma glucose and (A) ratios of the *Bacteroides*-*Prevotella* group to *C.coccoides*-*E.rectale* group, (B) relative abundance of the *Lactobacillus* group determined by SYBR Green qPCR assay in feces from human adults with type 2 diabetes (N = 18) and non-diabetic controls (N = 18). The Spearman Rank probability (P) and correlation (R) are shown in the graphs.

The total bacterial counts were similar in the diabetic and the control group having a median values of 10.6 (quartile ranges (QR) 10.1–11.3) and 10.5 (QR 10.0–11.3) log_10_ bacteria per g stool, respectively. The estimates of bacterial groups and genera by qPCR are presented in Figure 5. In diabetic group the median counts of *Bacteroides*-*Prevotella* group (10.2, QR 9.2–10.6), genus *Prevotella* (8.8, QR 7.6–11.5) and *C. leptum* subgroup (9.6, QR 9.0–9.7) were slightly but not significantly increased compared to the controls by approximately 0.7 log_10_ bacteria. Genus *Prevotella* was assessed in 25 (14 diabetic subjects and 11 controls) out of 36 samples analysed, whilst for the remaining samples it was below the detection limit of the applied qPCR assay. The difference in median counts of *C. coccoides*-*E. rectale*, *C. coccoides* and *Lactobacillus* groups, and genus *Roseburia* between the diabetic persons and controls was less than 0.2 log_10_ bacteria per g stool. The estimates of *Prevotella* and *Bacteroides*-*Prevotella* group were in some qPCR assays higher that the counts of total bacteria. The explanation might be that the primers, used for quantification of total bacteria, were not universal enough to amplify all bacterial populations, or alternatively that the group-specific primers amplified other targets as well. To overcome this limitation and to assess the differences between the groups of subjects we used ratios of bacterial counts. The ratios of *Bacteroides*-*Prevotella* to *C. coccoides*-*E. rectale* group correlated significantly and positively (R = 0.38; P = 0.03) with plasma glucose (Figure 6A) but not with BMI (R = 0.06, P = 0.71). Likewise, a positive correlation with plasma glucose was found for the ratios of *Bacteroides*-*Prevotella* to *C. coccoides* subgroup (R = 0.46, P<0.01; data nor shown) and for the proportion of the *Lactobacillus* group (R = 0.33, P = 0.05; Figure 6B). Higher ratios were in general related to a reduction in the *C. coccoides*-*E. rectale* and *C. coccoides* groups.

## Discussion

In this study we hypothesized that intestinal microbiota in humans with type 2 diabetes is different from non-diabetic persons. The hypothesis was tested on adults with a broad range of ages and BMIs, using pyrosequencing of the V4 region of the 16S rRNA gene and qPCR. To our knowledge no related studies on humans with diabetes type 2 have been published so far.

We demonstrated in this research that type 2 diabetes is associated with compositional changes in the intestinal microbiota mostly apparent at phylum and class levels. The relative abundance of *Firmicutes* was significantly lower, while the proportion of *Bacteroidetes* and *Proteobacteria* was somewhat higher in diabetic persons compared to their non-diabetic counterparts. Accordingly, the ratios of *Bacteroidetes* to *Firmicutes* significantly and positively correlated with reduced glucose tolerance. Assuming that diabetes and impaired glucose tolerance are linked to obesity, our results are in agreement with the recent evidence obtained for overweight persons by Schwiertz and colleagues [14], though contradict with other studies [11]. Furthermore, based on the assumption above, a positive correlation between ratios of *Bacteroidetes* to *Firmicutes* and BMI could be expected. However, the reverse tendency was observed (Figure 3D), indicating that overweight and diabetes are associated with different groups of the intestinal microbiota.

Bacterial groups that distinguished the diabetic from the non-diabetic microbiome included *Bacteroides*-*Prevotella* group versus class *Clostridia* and *C. coccoides*-*E.rectale* group, which ratios were significantly higher in diabetic persons. These results are supported by previous studies showing reduction in *Bacteroides*-*Prevotella* spp. related to a strong decrease of metabolic endotoxemia and inflammation in type 2 diabetes mice [6]. Accordingly, a significant reduction in *Clostridium* ssp, *C. coccoides* and an increase in the *Bacteroides*-*Prevotella* group along with body weight loss have been observed in human studies [16], [17]. The significantly higher levels of *Bacilli* and the *Lactobacillus* group in diabetic subjects compared to controls in the present study, have recently been reported in relation to type 2 diabetes in mice models [6] and to obesity in human adults [17], [18]. Genus *Lactobacillus* represents a heterogeneous group with well documented immunomodulating properties [19] and might potentially contribute to chronic inflammation in diabetic subjects.

The tendency of increased Chao1 diversity concurrently with BMI observed in this study, might be related to the negative correlation between BMI and *Bacteroidetes/Firmicutes* ratios (Figure 3D) as *Firmicutes* is a highly diverse division [12]. This observation is, however, in disagreement with recently published data on the obese twin pairs showing reduced bacterial diversity in obese individuals [11]. The reduced individual variation in diversity of the fecal microbiota observed in the diabetic group compared to the controls probably reflected the differences in diet, lifestyle or other factors [20] which are not possible to specify in the present study.

In an obesity study, using mice models, Cani and coworkers [21] proposed a hypothesis connecting metabolic diseases with the presence of Gram-negative bacteria in the gut, also offering a likely explanation of the differences between the diabetic and non-diabetic microbiomes in this study. The intestinal microbiota across the subjects with type 2 diabetes was relatively enriched with Gram-negative bacteria, belonging to the phyla *Bacteroidetes* and *Proteobacteria*. The main compounds of outer membranes in gram-negative bacteria are lipopolysaccharides (LPS), known as potent stimulators of inflammation, which can exhibit endotoxaemia [22]. Consequently, LPS will continue to be produced within the gut, which might trigger an inflammatory response and play a role in the development of diabetes.

In conclusion, our data suggest that the levels of glucose tolerance or severity of diabetes should be considered while linking microbiota with obesity and other metabolic diseases in humans. It is especially important for developing the strategies to modify the gut microbiota in order to control metabolic diseases, since obesity and diabetes might be associated with different bacterial populations.

## Methods

### Subjects and Sample Collection

The study protocol was approved by the Ethical Committee of Copenhagen and Frederiksberg Municipalities (KF 01-320695) and performed according to the declaration of Helsinki. Both written and verbal consent was obtained from the subjects of the study. The study included 36 males, diagnosed with type 2 diabetes (N = 18) or as being non-diabetic (N = 18) by their general practitioner confirmed by OGTT. The OGTT included the measurements of plasma glucose (mmol/l) at baseline and two hours after administration of 75 g glucose diluted in 500 ml of water. The BMIs were calculated from the formula: weight (kg)/height (m)^2^. Subjects' ages, BMIs (kg/m^2^) and 2-hour plasma glucose concentrations are presented in Table 1. Fecal samples were kept at 5°C immediately after defecation, brought to the laboratory within 24 hours and stored at −80°C before analysis.

### Extraction of Bacterial DNA from Fecal Samples

Total bacterial DNA was extracted from the fecal samples using the QIAamp DNA Stool Mini kit (QIAGEN, GmbH, Germany) according to the manufacturer's protocol for pathogen detection with slight modifications [23]. DNA concentration and quality in the extracts was determined by agarose gel electrophoresis (1% wt/vol agarose in Tris-acetate-EDTA (TAE) buffer) and with a NanoDrop 1000 spectrophotometer Thermo Scientific (Saveen Werner ApS, Denmark).

### Pyrosequencing

Tag-encoded amplicon pyrosequencing of fecal DNA was conducted for 10 persons with type 2 diabetes and 10 controls, matching in BMI and age. A selection criterion for diabetic persons was high severity of diabetes as evaluated by OGTT plasma glucose (Table 1). The primers used for pyrosequencing were a modified 530F-mod (GCCAGCMGCNGCGGTA; [24]) and 1061R (CRRCACGAGCTGACGAC; [25]) amplifying a 562 bp DNA fragment flanking the V4, V5 and V6 regions of the 16S rRNA gene ([26]; see SI Table S1). Modification of the primer 530F included two bases (TA) added to 3′-end in order to increase primer specificity. The sequence coverage of the forward and reverse primers was tested using the Probe Match feature at the Ribosomal Database Project release 10 (RDP 10; http://rdp.cme.msu.edu; [27]). The DNA concentration was measured with a NanoDrop spectrophotometer and adjusted to 5 ng/*µ*l for all samples. PCR amplification (in a volume of 40 *µ*l) was performed using 1x Phusion HF buffer, 2.5 mM magnesium chloride, 0.2 mM dNTP mixture, 0.8 U Phusion Hot Start DNA Polymerase (Finnzymes Oy, Espoo, Finland), 0.5 *µ*M of each primer (TAG Copenhagen A/S, Denmark) and 1 *µ*l diluted DNA sample. PCR was performed using the following cycle conditions: an initial denaturation at 98°C for 30 s, followed by 30 cycles of denaturation at 98°C for 5 s, annealing at 53°C for 20 s, elongation at 72°C for 20 s, and then a final elongation step at 72°C for 5 min. The PCR products were run on an agarose gel and purified using the QIAEX II Gel Extraction Kit (QIAGEN). Second round of PCR was performed as described above, except that the primers with adapters and tags were used, the number of cycles was reduced to 10 and the annealing temperature was increased to 56°C. Addition of adapter and tags specific to each sample was done using the custom primers with adapter A and 10 tags (see SI Table S1) required for pyrosequencing [24]. The concentration of the tagged PCR product was determined by qPCR. The standard DNA used in qPCR was prepared from *Pseudomonas putida* and quantified against a standard concentration of 200 base ssDNA oligo (TAG Copenhagen A/S, Denmark). The qPCR was performed in a 25 *µ*l volume, using 1x Brilliant buffer (Stratagene, Cedar Creek, Texas, USA), 0.5 µM of each primer FLX (forward: GCCTCCCTCGCGCCATCAG and reverse: GCCTTGCCAGCCCGCTCAG) and 1 *µ*l of diluted PCR products with tags and adapters. Amplification was conducted using a Mx-3000 thermocycler (Stratagene) at the following conditions: an initial denaturation at 95°C for 10 min, followed by 40 cycles of denaturation at 95°C for 30 s, annealing at 60°C for 60 s. Amplicons were then mixed in approximately equal concentration (5×10^7^ copies per µl) to ensure equal representation of each sample. A two-region 454 sequencing run was performed on a GS FLX Standard PicoTiterPlate (70X75) by using a GS FLX pyrosequencing system according to the manufacturer's instructions (Roche).

Analysis of sequencing data was conducted using Pyrosequencing Pipeline tools at RDP 10 (http://pyro.cme.msu.edu/index.jsp). The RDP Classifier was used to assign 16S rRNA gene sequences to taxonomical hierarchy with a confidence threshold of 80%. Bacterial diversity was determined by sampling-based analysis of operational taxonomic units (OTUs) and showed by rarefaction curves. Comparison of bacterial richness across the samples was performed by Chao1 estimate at the distance of 3%, usually applied to characterize richness at species level [12], [28]. As the Chao1 estimates are dependent on the size of the sequence libraries, the sample sizes from the different subject we equalized by random subtraction.

### Real-Time qPCR

Bacterial groups in fecal samples from 18 subjects with type 2 diabetes and 18 controls were quantified by qPCR using the 7500 Fast Real-time PCR System (Applied Biosystems, USA) and the primers shown in Table 3 (TAG Copenhagen A/S, Denmark). Genus-specific primers for amplification of *Prevotella* and *Roseburia* were designed using 16S rRNA gene sequences from the RDP 10. Sequences were aligned by the ClustalW software provided by the European Bioinformatics Institute (http://www.ebi.ac.uk/Tools/clustalw2/index.html). The target-specific sites were assessed by “Oligo” Primer Analysis Software version 6.71 (Molecular Biology Insights, Inc., USA). Specificity of the primers was evaluated *in silico* using the nucleotide BLAST, blastn algorithm (http://blast.ncbi.nlm.nih.gov/Blast.cgi). *Prevotella*-specific primers were targeting *Prevotella falsenii*, *P. copri*, *P. nigrescens*, *P. intermedia*, *P. pallens*, *P. maculosa* and *P. oris*. Primers targeting *Roseburia* were specific for species *Roseburia faecis*, *R. hominis*, *R. intestnalis* and *R. cecicola*.

**Table 3:** Primers used in the study for real-time qPCR and size of PCR products.

Target organism	Primer a	Sequence (5′to 3′)	PCR productbp	Reference
*Clostridium coccoides*-*Eubacteria rectale* group	ClEubF	CGGTACCTGACTAAGAAGC	429	[31]
	ClEubR	AGTTTYATTCTTGCGAACG		
*Clostridium leptum* subgroup	CleptF	GCACAAGCAGTGGAGT	239	[32]
	CleptR	CTTCCTCCGTTTTGTCAA		
*Clostridium coccoides* subgroup	CcocF	AAATGACGGTACCTGACTAA	440	
	CcocR	CTTTGAGTTTCATTCTTGCGAA		
*Roseburia* b	RosF	TACTGCATTGGAAACTGTCG	230	This study
	RosR	CGGCACCGAAGAGCAAT		
*Bacteroides*-*Prevotella* group	BacF	GAAGGTCCCCCACATTG	410	[33]
	BacR	CAATCGGAGTTCTTCGTG		
*Prevotella* c	PrevF	CACCAAGGCGACGATCA	283	This study
	PrevR	GGATAACGCCYGGACCT		
*Lactobacillus* group	LacF	AGCAGTAGGGAATCTTCCA	341	[34]
	LacR	CACCGCTACACATGGAG		
*Bifidobacterium*	BifF	GCGTGCTTAACACATGCAAGTC	126	[35]
	BifR	CACCCGTTTCCAGGAGCTATT		
All bacteria	UnivF	TCCTACGGGAGGCAGCAGT	466	[36]
	UnivR	GACTACCAGGGTATCTAATCCTGTT		

a Primers F (forward) and R (reverse) targeting the 16S rRNA gene.

b Primers targeting *Roseburia* species: *R. faecis*, *R. hominis*, *R. intestnalis* and *R. cecicola*.

c Primers targeting *Prevotella* species: *P. falsenii*, *P. copri, P. nigrescens, P. intermedia, P. pallens, P. maculosa* and *P. oris*.

The qPCR reaction mixture (20 *µ*l) was composed of 0.3 µM of each universal primer or 0.5 µM of each specific primer, 1x Power SYBR Green PCR Master Mix (Applied Biosystems, Calif., USA), and 4 *µ*l fecal DNA added in 10-fold serial dilutions starting from 100-fold dilution to diminish the effect of inhibitors. The amplification program consisted of one cycle of 95°C for 10 min, followed by 40 cycles of 95°C for 15 s, and 60°C for 1 min. Standard curves were constructed for each experiment using 10-fold serial dilutions of bacterial genomic DNA of known concentration. Genomic DNA from *L. acidophilus* NCFM (ATCC 700396) and from *Bifidobacterium animalis subsp. lactis (B. lactis) Bi-07 (ATCC SD5220)* was extracted with the use of GenElute Bacterial Genomic DNA Kit (Sigma-Aldrich, Germany) according to the manufacture's instructions. DNA from *Roseburia intestinalis* DSM14610, *Prevotella copri* DSM18205, *Clostridium leptum* DSM 753, *Clostridium coccoides* DSM935, *Clostridium nexile* DSM1787 and *Bacteroides thetaiotaomicron* DSM 2079 was purchased from DSM collection (Deutsche Sammlung von Mikroorganismen und Zellkulturen GmbH, Germany). Standard curves were created according to Applied Biosystems tutorials (http://www3.appliedbiosystems.com) and normalized to the copy number of the 16S rRNA gene for each species. For the species which copy number of 16S rRNA operon was not published, it was calculated by averaging the operon numbers of the closest bacterial taxa from the ribosomal RNA database rrnDB (http://ribosome.mmg.msu.edu/rrndb/index.php; [29]). Cell numbers of bacteria in fecal samples were calculated from the threshold cycle values (Ct) and expressed as quantity of bacteria per gram feces [30].

### Statistical Analysis

PCA plots were generated by Matlab 2008b using in-house algorithms (http://www.mathworks.com). Differences in bacterial populations between the diabetic and control groups were assessed using the two-sided Wilcoxon Rank Sum test (Statistics Online Computational Resource (SOCR), http://www.socr.ucla.edu/SOCR.html). Correlation between the variables was computed by Spearman Rank correlation provided by Free Statistics Software (version 1.1.23-r4, Office for Research Development and Education, http://www.wessa.net/). The qPCR results were graphically presented by Box and Whisker charts (Microsoft Office Excel 2007) and expressed as medians with quartile ranges (QR).

## Supporting Information

Figure S1 Relative abundances of bacterial genera. Relative abundances (%) of bacterial genera in feces from human adults with type 2 diabetes (green triangles, N = 10) and non-diabetic controls (blue dots, N = 10) determined by pyrosequencing of the V4 region of the 16S rRNA gene. Mean values are denoted by red crosses and numbers. Values out of scale are shown in brackets.
(0.28 MB TIF)

Figure S2 PCA plots of bacterial families and genera. PCA plots showing the grouping of human adults with type 2 diabetes (triangles, D1-D10) and non-diabetic controls (dots, C1-C10) according to the abundances of bacterial families (A) and genera (B) in fecal bacterial DNA as determined by pyrosequencing of the V4 region of the 16s rRNA gene.
(0.32 MB TIF)

Table S1 Primers, adaptor and sample-specific barcodes used in the study for tag-encoded amplicon pyrosequencing of the V4 region of the 16S rRNA gene.
(0.03 MB DOC)
