# Supplementary figures and images for: Gut Microbiota in Human Adults with Type 2 Diabetes Differs from Non-Diabetic Adults
Source: PLoS One. 2010 Feb 5;5(2):e9085. doi: 10.1371/journal.pone.0009085 (PMC2816710; doi:10.1371/journal.pone.0009085)

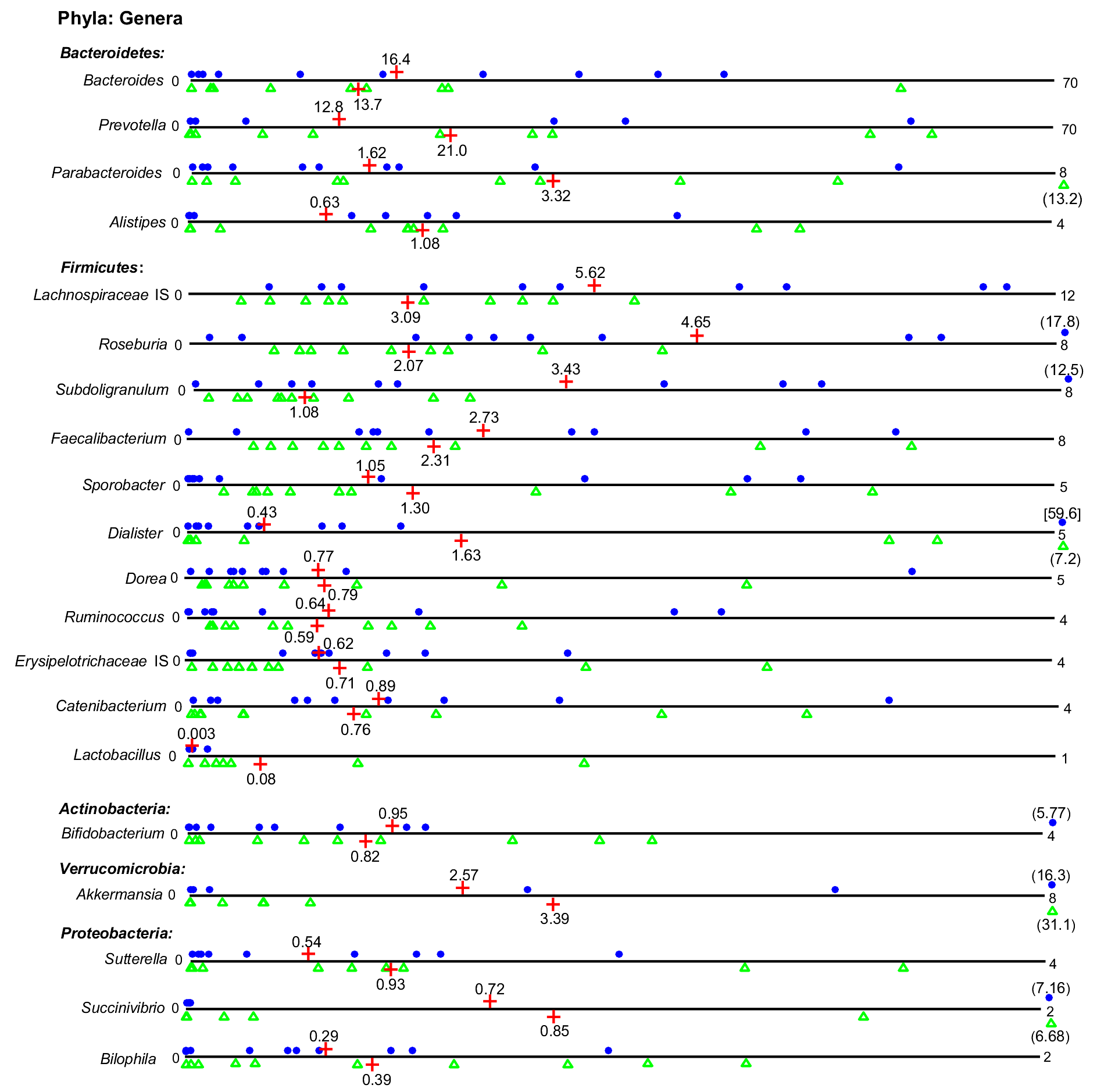

Supplement: Figure S1 — Relative abundances of bacterial genera. Relative abundances (%) of bacterial genera in feces from human adults with type 2 diabetes (green triangles, N = 10) and non-diabetic controls (blue dots, N = 10) determined by pyrosequencing of the V4 region of the 16S rRNA gene. Mean values are denoted by red crosses and numbers. Values out of scale are shown in brackets. (0.28 MB TIF) [file pone.0009085.s001.tif]

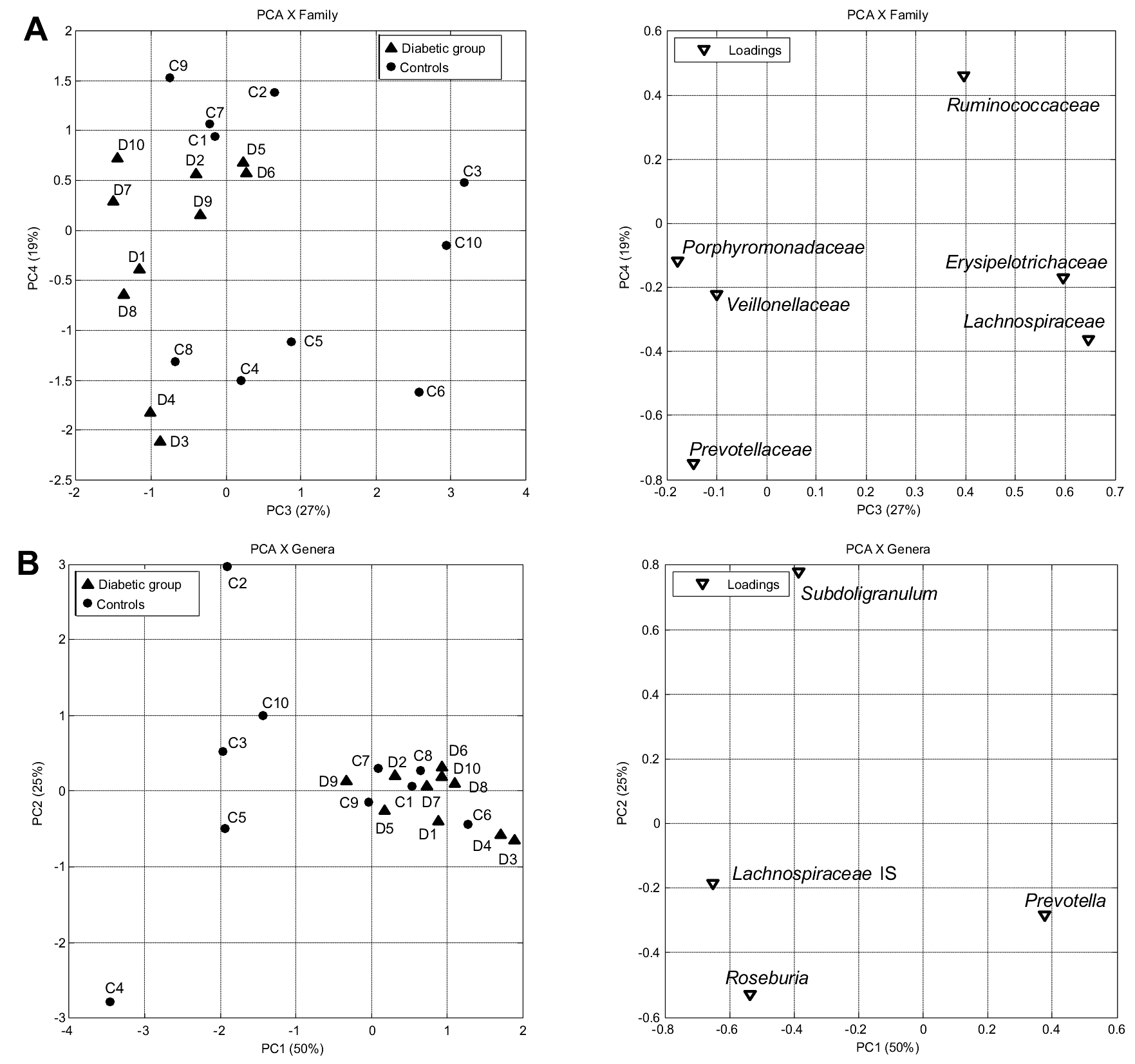

Supplement: Figure S2 — PCA plots of bacterial families and genera. PCA plots showing the grouping of human adults with type 2 diabetes (triangles, D1-D10) and non-diabetic controls (dots, C1-C10) according to the abundances of bacterial families (A) and genera (B) in fecal bacterial DNA as determined by pyrosequencing of the V4 region of the 16s rRNA gene. (0.32 MB TIF) [file pone.0009085.s002.tif]
