# Supplementary material for: Gut Microbiota in Human Adults with Type 2 Diabetes Differs from Non-Diabetic Adults
Source: PLoS One. 2010 Feb 5;5(2):e9085. doi: 10.1371/journal.pone.0009085 (PMC2816710; doi:10.1371/journal.pone.0009085)
Supplement: Table S1 — Primers, adaptor and sample-specific barcodes used in the study for tag-encoded amplicon pyrosequencing of the V4 region of the 16S rRNA gene. (0.03 MB DOC) [file pone.0009085.s003.doc]

| Primer | Adaptor | Barcode ^a^ | Primer sequence ^b^ |
| --- | --- | --- | --- |
| 530F-mod | GCCTTGCCAGCCCGCTCAG | CGCACTACGTGT | GCCAGCMGCNGCGGTA |
|  |  | CGCATACAGTGT |  |
|  |  | CGCATCTATAGT |  |
|  |  | CGCCAGAAAAGT |  |
|  |  | TGTGACGTACGT |  |
|  |  | TGTGTGCATAGT |  |
|  |  | TGTGCATCACGT |  |
|  |  | TGTGCCTAGAGT |  |
|  |  | TGTACATAGTGT |  |
|  |  | TGTACATTGAGT |  |
| 1061R |  |  | CRRCACGAGCTGACGAC |

^a^ Sample-specific barcodes

^b^ Primers have degenerated nucleotides positions designated as follows: M = A or C; N = A, T, C or G; R = A or G
